# Supplementary material for: Knowledge on Cervical Cancer Services and Associated Risk Factors by Health Workers in the Eastern Cape Province
Source: Healthcare (Basel). 2023 Jan 21;11(3):325. doi: 10.3390/healthcare11030325 (PMC9914601; doi:10.3390/healthcare11030325)
Supplement: Supplementary file 1 [file healthcare-11-00325-s001.zip › healthcare-2063857-supplementary.pdf]

Knowledge on Cervical Cancer and associated risk factors by Health Workers in the Eastern Cape Province

Supplementary Tables (ST)

ST1: Risk Factors for cervical cancer.

Items related cervical cancer risk factors		№	%
Are you more likely to get cervical cancer if someone in your family, has it?	Yes	78	72.2
	No	30	27.8
Is smoking a risk factor for cervical cancer?	Yes	76	70.4
	No	32	29.6
Is oral contraception a risk factor of cervical cancer?	Yes	65	60.2
	No	43	39.8
Is giving birth to many babies a risk factor for cervical cancer?	Yes	73	67.6
	No	35	32.4
Is having many different sexual partners a risk factor for cervical cancer?	Yes	93	86.1
	No	15	13.9
Is Pap Smear an effective treatment for cervical cancer?	Yes	33	30.6
	No	75	69.4
Is Pap Smear an effective diagnosis for cervical cancer?	Yes	33	30.6
	No	75	69.4
What Pap smear is used for?	Detects cervical cancer cell changes	53	49.1
	Other answers	55	50.9
Which of these are common signs or symptoms of cervical cancer?	Longer or heavier periods	85	78.7
	Abnormal vaginal bleeding between periods	97	89.8
	Pain or bleeding during sex	89	82.4
	Unusual or smelling vaginal discharge which does not go away	93	86.1
	Unexplained lower backpain	38	35.2
	Increased urination	64	59.3

ST2: Relationship between location and cervical cancer knowledge of the participants.

Location	According to the SA screening policy, how many times can a woman be screened?			Is there any available vaccine for cervical cancer?			What is the recommended age for cervical cancer related vaccine?			What is the recommended age for cervical cancer screening?		
	Three times	Other	Total	Yes	No	Total	9 - 12	Other	Total	>30 yrs	Other	Total
Baziya CHC	3	14	17	12	5	17	15	2	17	10	7	17
Isimela Hospital	7	9	16	7	9	16	4	12	16	7	9	16
Dr Malizompehle Gateway Clinic	0	9	9	8	1	9	6	3	9	4	5	9
Mthatha Gateway Clinic	0	13	13	7	6	13	7	6	13	9	4	13
Mbekweni CHC	4	9	13	12	1	13	11	2	13	7	6	13
Mhlakulo CHC	5	1	6	6	0	6	6	0	6	4	2	6
Mntwana Clinic	0	6	6	4	2	6	4	2	6	4	2	6
Mount Frere Gateway Clinic	0	11	11	8	3	11	5	6	11	7	4	11
Mqanduli CHC	0	1	1	1	0	1	1	0	1	1	0	1
Qumbu CHC	0	15	15	11	4	15	10	5	15	6	9	15
ST Barnabas Gateway Clinic	0	1	1	1	0	1	1	0	1	0	1	1
Total	19	89	108	77	31	108	70	38	108	59	49	108

ST3: Relation between occupation and cervical cancer screening knowledge.

Occupation	According to the SA screening policy, how many times can a woman be screened?			Is there any available vaccine for cervical cancer?			What is the recommended age for cervical cancer related vaccine?			What is the recommended age for cervical cancer Screening?		
	Three times	Other	Total	Yes	No	Total	9 - 12	Other	Total	>30 yrs	Other	Total
Registered nurse	19	78	97	70	27	97	64	33	97	56	41	97
Enrolled nurse	0	11	11	7	4	11	6	5	11	3	8	11
Total	19	89	108	77	31	108	70	38	108	59	49	108

ST4: Relation between length of practice and cervical cancer score knowledge.

**Length of practice (years)**	**Score Knowledge Category**		
	**Inadequate**	**Adequate**	**Total**
1 – 5	22	11	33
6 – 10	31	12	43
> 10	22	10	32
Total	75	33	108
